# Renal dialysis and long-term treatment of a dog with kidney disease associated with canine leishmaniosis

**DOI:** 10.1186/s13071-018-2719-6

**Published:** 2018-03-20

**Authors:** Gad Baneth, Gilad Segev, Michal Mazaki-Tovi, Hila Chen, Sharon Kuzi

**Affiliations:** Koret School of Veterinary Medicine, The Hebrew University of Jerusalem, Rehovot, Israel

**Keywords:** Canine leishmaniosis, Epistaxis, Hemodialysis, Acute kidney injury, Chronic kidney disease

## Abstract

**Background:**

Renal disease is considered the main cause of natural mortality in dogs with canine leishmaniosis. The pathological mechanisms associated with kidney injury in canine leishmaniosis include immune complex glomerulonephritis, tubulointerstitial nephritis and occasionally renal amyloidosis. Proteinuria is a frequent finding in canine leishmaniosis and its quantification by the urine protein-creatinine ratio (UPC) is an important parameter in the staging of canine lesihmaniosis as presented by the LeishVet group.

**Results:**

A 4.5 year-old spayed female Belgian Malinois dog was presented to the Hebrew University Veterinary Teaching hospital with epistaxis and rhinitis and diagnosed also with proteinuria and acute kidney injury (AKI IRIS grade V) associated with canine leishmaniosis that developed to LeishVet stage III with chronic kidney disease (CKD) after stabilization. Clinicopathologic abnormalities included azotemia with a peak creatinine concentration of 7.76 mg/dl (reference interval, 0.3–1.2 ng/dl), hypoalbuminemia (1.76 g/dl, reference interval 3-4.4 g/dl), hyperglobulinemia (4.54 g/dl, reference interval 1.8–3.9 g/dl) and proteinuria (urine protein/creatinine ratio 15.6, normal < 0.2). Serology by the enzyme-linked immunosorbent assay (ELISA) for *Leishmania infantum* was positive with high antibody levels. The dog was hospitalized and treated with intermittent hemodialysis, feeding through an esophageal feeding tube, medical treatment for protein losing nephropathy and antileishmanial treatment with allopurinol. Kidney function gradually improved and the dog’s creatinine levels and proteinuria decreased until complete normalization two years after the acute insult. However, rhinitis and sneezing persisted and although the anti-leishmanial antibodies decreased over time, the dog remains constantly seropositive.

**Conclusions:**

To our knowledge, this is the first report of hemodialysis management of AKI associated with canine leishmaniosis. Hemodialysis was imperative in stabilizing the dog’s renal disease and controlling its azotemia. It demostrates that hemodialysis can be beneficial in the management of acute deterioration of kidney disease in canine leishmaniosis.

**Electronic supplementary material:**

The online version of this article (10.1186/s13071-018-2719-6) contains supplementary material, which is available to authorized users.

## Background

Renal injury is common in canine leishmaniosis and ranges from sub-clinical to severe and fatal end-stage kidney disease. Although dermal manifestations and lymphadenomegally are the most common manifestations of canine leishmaniosis, renal disease is considered the main cause of mortality in dogs with this disease and it occasionally presents without typical skin abnormalities [1, 2]. The pathological mechanisms associated with kidney injury in canine leishmaniosis include the deposition of immune complexes with glomerulonephritis which can be mesangioproliferative or membranoproliferative, tubulointersitital nephritis and occasionally renal amyloidosis. Proteinuria is a frequent finding in canine leishmaniosis and its quantification by the urine protein-creatinine ratio (UPC) is an important parameter in the staging of the chronic kidney disease as well as canine leishmaniosis as presented by the LeishVet group, in addition to other markers of disease severity [1, 3–6].

Acute kidney injury (AKI) leading to severe uremia, regardless of its underlying cause, is associated with high morbidity and mortality [7–9]. Most dogs with AKI are managed medically, but when the disease is severe, the clinical and pathologic consequences of AKI may no longer be managed effectively with conventional medical therapy. Hemodialysis, initially developed for management of human kidney disease, is an advanced extracorporeal renal replacement therapy for uremic and overhydrated patients that has been adapted to canine and feline medicine. It has the capability to remove uremic toxins, correct fluid and electrolyte disorders, restore acid-base balance, and sustain the life of the patient until the kidney injury is repaired [9]. As a consequence, hemodialysis extends life expectancies of patients with severe uremia and expands the window of opportunity for recovery and the potential for a favorable outcome. Nonetheless, the mortality of canine patients with AKI managed with hemodialysis remains as high as 50% and this treatment is costly [9]. Hemodialysis requires specialized equipment and expertise during and in-between treatments and is currently carried out mainly in major veterinary referral centers by specially trained staff.

The aim of this report is to describe the management and follow up of a dog with canine leishmaniosis and associated kidney disease by medical treatment and hemodialysis. To the best of our knowledge, this is the first report of the use of hemodialysis in the treatment of canine leishmaniosis.

## Methods

### Case report

A 4.5 year old spayed female Belgian Malinois dog was presented to a veterinary clinic in Central Israel with complaints of episodic right nostril epistaxis initially seen by the owners 10 weeks prior to presentation. Blood tests which included a complete blood count (CBC), serum biochemistry panel and a blood coagulation panel were within normal limits. Rhinoscopy performed at the referring veterinerian’s clinic showed that the right nasal mucosal surfaces were thickened and edematous and an ulcer was noted on the dorsal meatus, while the left side of the nose appeared normal. The dog was referred to the Hebrew University Veterinary Teaching hospital (HUVTH) for further evaluation. Physical examination was unremarkable and results of the CBC, serum biochemisty and coagulation tests consisting of prothrombin time (PT), partial thromboplastin time (PTT) and buccal mucosal bleeding time (BMBT) were within normal limits (PT < 12 s; PTT < 15 s; BMBT < 4 min, range 2–5 min) except for the BMBT which was prolonged (5 min). Computed tomography (CT) of the nasal cavity showed bilateral thickening of the caudal nasal cavity conchal walls without obvious bone involvement, and accumulation of fluid in the cranial nasal cavity. A second rhinoscopy was performed and biopsies were taken for histopathology. Lethargy, inappetance and severe nasal bleeding were noted by the owners 3 days following rhinoscopy and biopsies. Epistaxis was unresponsive to local treatment with adrenalin, sedation with acepromazine (Medimarket, Netanya, Israel) at 0.05 mg/kg IM and gelfoam (Mascia Brunelli, Milan, Italy) placement, finally necessitating surgery with bilateral carotid ligation. The dog was discharged without further bleeding and with a normal serum creatinine (0.5 mg/dl; reference range 0.3–1.2 mg/dl) 2 days following surgery, with borad spectrum antibiotic treatment against potential bacterial infection of the surgical sites (amoxicillin/clauvalonic acid 25 mg/kg q 12 h, Smithkline Beecham, Brentford, UK). Biopsy results indicated lymphocytic eosinophilc rhinitis with no evidence of bacterial or fungal infection. Three weeks after surgery, the dog was admitted after 2 days of decreased appetite and vomiting. It had increased episodes of sneezing and stretor, and nasal secretions had reappeared. Blood tests revealed mild normocytic-normochromic anemia (hematocrit 34, reference range 37.1–57%) with leukocytosis of 19 × 10^3^/mm^3^ (reference range 5.2–13.9 × 10^3^/mm^3^), azotemia with elevated creatinine (3.1 mg/dl) and urea (81 mg/dl; reference range 10.7–53.5 mg/dl), hypoalbuminemia (1.76 g/dl; reference range 3–4.4 g/dl), hyperglobulinemia (4.54 g/dl; reference range 1.8–3.9 g/dl) and an albumin/globulin ratio of 0.39 (normal > 0.7). Urinalysis indicated a high magnitude of proteinuria (+4 by stick) and urine protein/creatinine ratio (UPC) of 15.2 (normal < 0.2). The dog was diagnosed with AKI and hopitalized for fluid and medical treatment and monitoring. Serology for leptospirosis, was negative using the microscopic agglutination test (MAT). At this stage, due to the hyperglobulinemia, proteinuria and kidney injury it was suspected for canine leishmaniosis and tested by an ELISA quantitative serology using *Leishmania infantum* antigen as previously described [10]. *Leishmania* serology was highly positive with an optical density (OD) of 1.6 (cut off level 0.3) and treatment against leishmaniosis was started with allopurinol (Dexcel Pharma, Or Akiva, Israel) at 10 mg/kg q 12 h PO. Despite fluid and medical therapy, the dog’s azotemia worsened within the next 3 days (creatinine increased to 7.8 mg/dl, urea to 200 mg/dl and phosphorous to 15 mg/dl, reference interval 3.0–6.2 mg/dl), reaching a state of AKI International Renal Interst Society (IRIS) grade V [11]. Hemodialysis was initiated to decrease azotemia and allow the kidneys time to recover.

Hemodialysis was performed using routine technique as previously described [12]. Briefly, a double lumen 11.5 Fr (French), 24 cm double lumen catheter was asepticly inserted to the right jugular vein. Dialysis treatment was delivered using the AK-200S dialysis delivery system (Gambro Renal Products, Lund, Sweden) using a pediatric extracorporeal circuit (Gambro Renal Products, Lund, Sweden) with priming volume of 70 mls and the FX60 dialyzer (Fresenius Medical Care, Tel Aviv, Israel) with a priming volume of 74 mls. A total of 3 dialysis treatments of 4 h duration were performed over 8 days. Dialysis treatments were discontinued thereafter as kidney function improved. An esophageal feeding tube was placed surgically through which water, food and medication were adminstered.

The dog was discharged after 20 days of hospitalization at the HUVTH with a creatinine of 2.4 mg/dl. Treatment at home included allopurinol (10 mg/kg q 12 h PO) for leishmaniosis, famotidine (West-Ward, Eatontown, NJ, USA) at 1 mg/kg q 24 h PO against gastric ulceration, the antibiotic amoxillin-caluvalonic acid (25 mg/kg q 12 h PO) against bacterial infection, and the antiemetics maroptinat citrate (Zoetis, Kalamazoo, MI, USA) at 1mg/kg q 24 h PO and metoclopramide (Rafa laboratories, Jerusalem, Israel) at 0.5 mg/kg q 8 h PO. Blood tests at a recheck 11 days after discharge revealed further improvemnt in kidney function (creatinine 1.9 mg/dl) and treatment with enalapril (Dexcel Pharma, Or Akiva, Israel) at 0.25 mg/kg q 12 h PO was started for decreasing proteinuria.

## Results

### Case report

A detailed clinical history followup of the dog during 15 weeks after its discharge from the hospitalization with hemodialysis is included in Additional file 1: Table S1. Two years after hemodialysis, when writing this report, the dog is still being monitored and treated medically for chronic kidney disease (CKD) and is currently at IRIS CKD Stage I, non-proteinuruic, non-hypertensive [11]. It remains seropositive for *L. infantum* antigen by ELISA, although with a lower antibody level compared to its initial testing (0.73 OD 21 months after allopurinol treatment initiation), despite continuous treatment with allopurinol and a course of miltefosine (Virbac, Carros, France) at 2 mg/kg q 24 h PO for 28 days, administered five months after discharge from hospital when the ELISA serology result was 1.32 OD. The dog is currently classified as having leishmaniosis LeishVet stage IIa [6], compared to LeishVet stage IV with creatinine levels above 5 mg/dl and UPC higher than 5 while kidney injury was most severe. The dog gained 6 kg since discharge, but despite marked clinical improvement, it continues to sneeze with occasional nasal mucous discharge due to chronic rhinitis but no apparent bleeding, and has episodes of appetite loss. Chronic rhinitis is treated with long term doxycycline (Dexcel, Or Akiva, Israel) at 10 mg/kg q 24 h PO for 28 days against secondary bacterial infection of the nasal cavity, which is repeated when nasal discharge increases. Low dose meloxicam (Boehringer Ingelheim, MO, USA) at. 0.01 mg/kg, q 24 h PO was further added to the treatment to ameliorate clinical signs associated with rhinitis, but discontinued due to a mild increase in serum creatinine concentration (to 1.3 mg/dl), which resolved after discontinuation.

## Discussion

This study describes the treatment and management of a dog with canine leishmaniosis associated with kidney disease. Hemodialysis was imperative in stabilizing the dog’s renal injury and controlling its azotemia. Although the dog did not completely recover from canine leishmaniosis despite continuous antileishmanial treatment, as indicated by its persistent positive serology to *L. infantum antigen* over two years of treatment, it was brought to a state of partial remission from severe disease.

This first report of hemodialysis management of kidney disease associated with canine leishmaniosis demostrates that hemodialysis can be beneficial in the management of renal disease associated with canine leishmaniosis and should be considered as an optional treatment in leishmaniosis with AKI.

Epistaxis has been described in studies of leishmaniosis in 6–10% of dogs reported with this clinical disease in Italy and Greece [13, 14]. The causes of epistaxis in canine leishmaniosis are multifactorial and include lymhoplasmocytic or pyogranulomatous rhinitis with or without nasal mucosa ulceration, thromobocytopenia, thrombocytopathy, hyperglobulinemia-induced serum hyperviscosity, and co-infections with pathogens such as *Ehrlichia canis* [2, 15, 16]. In a study by Juttner et al. [15], the nasal epithelium of ten dogs with canine leismaniosis whose nasal cavity was sampled at necropsy was examined by histopathology. All dogs showed areas of erosion and the nasal lamina propria had vascular congestion and focal hemorrhages with superficial perivascular inflammatory infiltrates of lymphocytes, plasma cells, neutrophils and macrophages with *Leishmania* amastigotes [15]. The findings of an ulcer in the nasal cavity of the dog described in the present study with lymphocytic eosinophilic rhinitis are in agreement with some of the histopahtological descriptions from the study of these ten dogs [15]. The reasons for the eosinophilc infiltration of the nasal mucosa in the dog reported here are unknown; nevertheless eosinophilic rhinitis has also been described in a previous study on epistaxis in canine leishmaniosis and in dogs with chronic idiopathic inflammatory rhinitis [16, 17]. The prolonged BMBT found in the dog described here may have been caused by thrombocytopathy associated with platelet binding by antibodies or by vasculitis which have both been described in canine leishmaniosis [18, 19]. Doxycycline combined with anti-inflammatory drugs, either non-steroidal anti-inflammatory drugs (NSAIDs) or steroids, are reported to have a benificial effect in non-specific, lymphocytic-plasmacytic rhintis in dogs [19]. In the current dog with leishmaniosis, secondary infection and inflammation may have contributed to the rhinitis, thus treatment with doxycycline was perscribed and was indeed followed by some improvement in the frequency and content of nasal discharge. Additinal anti-inflammatory drugs may worsen kidney injury (NSAIDs) or infection (steroids); and indeed, a short treatment with low dose meloxicam was followed by an increase in creatinine that was reversed after discontinuation.

Some degree of renal pathology can be found by histology in almost every dog with canine leishmaniosis; however, this does not always progress to decreased kidney function. A study of 55 seropositive dogs from Brazil found glomerulonephritis in all dogs, of which 13 were infected sub-clinically. Interstitial nephritis was found in 78% of the dogs, and glomerular deposition of parasite antigen in 91% [3]. In another study, 41 seropositive dogs from Italy were tested for proteinuria and also subjected to ultrasound-assisted renal biopsy for histopathologic evaluation. All dogs had proteinuria and glomerular lesions detected by histopathology, with 23 dogs (55%) showing interstitial or tubular lesions [4]. However, proteinuria has been reported to markedly decrease with normalization of the UPC in dogs treated successfully against leishmaniosis [20, 21].

The dog in this report is an example of chronic rhinitis and AKI which developed into CKD associated with canine leishmaniosis. Possible causes for the acute decrease in kidney function in this dog include untreated leishmaniosis and possibly decreased kidney perfusion due to dehydration (i.e. decreased water consumption) associated with rhinitis and anesthesia and blood loss during the dog’s first visit. Other common causes of canine AKI include leptospirosis which was ruled out by serology, hypoadrenocorticism which is frequently associated with electrolyte disorders, which were not found in this case, toxicities including ethylene glycol toxicity, which were not likely due to the dog’s history, ultrasonographic finding and recovery without specific antidote treatment, and nephrotoxic medications which were not administered prior to the event. It was initially classified as having LeishVet’s leishmaniosis stage IV and AKI IRIS grade V, and has improved with treatment to reach LeishVet stage IIa with CKD stage I [5, 6, 11]. This is in agreement with the notion that LeishVet’s staging is dynamic and may change over time due to improvement or deterioration in the dog’s clinical status.

## Conclusions

Hemodialysis management of kidney disease associated with canine leishmaniosis is described here for the first time in dogs. Management of AKI associated with canine leishmaniosis by hemodialysis and long term treatment of CKD were able to stabilize and prolong the life of the treated dog for more than two years. Therefore, hemodialysis is an important treatment modality that can be used, when available, for the management of kidney injury associated with leishmaniosis.

## Additional file

Additional file 1: **Table S1.** Follow up of clinical findings and treatment of the dog during 15 weeks after its discharge from the Hebrew University Veterinary Teaching Hospital. Earlier findings are described in the text. (DOCX 14 kb)

## Abbreviations

AKI: Acute kidney injury
CKD: Chronic kidney disease
CT: Computed tomography
IM: Intramuscular
MAT: Microscopic agglutination test
OD: Optical density
PO: *Per os*
UPC: Urine protein/creatinine ratio
