# Supplementary material for: Renal dialysis and long-term treatment of a dog with kidney disease associated with canine leishmaniosis
Source: Parasit Vectors. 2018 Mar 20;11:151. doi: 10.1186/s13071-018-2719-6 (PMC5859824; doi:10.1186/s13071-018-2719-6)
Supplement: Supplementary file 1 — Table S1. Follow up of clinical findings and treatment of the dog during 15 weeks after its discharge from the Hebrew University Veterinary Teaching Hospital. Earlier findings are described in the text. (DOCX 14 kb) [file 13071_2018_2719_MOESM1_ESM.docx]

**Additional file 1: Table S1** Follow up of clinical findings and treatment of the dog during 15 weeks after its discharge from the Hebrew University Veterinary Teaching Hospital. Earlier findings are described in the text.

| **Time from discharge after hospitalization with hemodialysis treatment** | **Clinical findings** | **Clinical-pathological parameters*** | **Treatment**** |
| --- | --- | --- | --- |
| Ten days | Owners report of improvement in the dog's well-being. Body weight 19 kg. | Creatinine 1.9 mg/dL; Albumin 1.4 g/dL;  PCV 35% | Dog gets water and medication through esophageal tube. Allopurinol; famoitidine; maropitante citrate; enalapril |
| Three weeks | Four days of decreased appetite, sneezing and secretions from right nostril. Body weight 20 kg. | Creatinine 1.3 mg/dL; albumin 1.32 g/dL; phosphorous 5.89 mg/dL; UPC 20.3; PCV 34% | As above with the addition of mirtazapine for appetite enhancement |
| Four weeks | Improvement in appetite | Creatinine 1.2 mg/dL; PCV 34%; UPC 10 | As above |
| Five weeks | Improved appetite. Continued sneezing and nasal secretions. Body weight 21 kg. | Creatinine 0.5 mg/dL | As above |
| Seven weeks |  | Creatinine 0.7; albumin 1.33 g/dL; UPC 4 | As above |
| Ten weeks | Increase in appetite. | Creatinine 0.8 mg/dL; albumin 2.02 g/dL | As above. Started being fed with a kidney diet (K/d Hills) |
| Twelve weeks | Good appetite. Continued sneezing and nasal secretions. | Creatinine 1.0 mg/dL; albumin 2.07 g/dL; UPC 0.35 | As above |
| Fifteen weeks | Good appetite. Continued sneezing and occasional moderate nasal secretions. Body weight 23.8 kg | Creatinine 1.1 mg/dL, albumin 2.6 g/dL; globulins 4.0 g/dL; phosphorous 4.76 mg/dL. Negative fungal and pathogenic bacteria cultures from nasal swabs. | As above |

* Reference ranges for the clinic-pathological analyts are included in the text.

** Drug doses and manufacturer details are included in the text.
